# The Effectiveness of an Educational Game for Teaching Optometry Students Basic and Applied Science

**DOI:** 10.1371/journal.pone.0156389

**Published:** 2016-05-27

**Authors:** Richard Trevino, Carolyn Majcher, Jeff Rabin, Theresa Kent, Yutaka Maki, Timothy Wingert

**Affiliations:** Rosenberg School of Optometry, University of the Incarnate Word, San Antonio, Texas, United States of America; University of Westminster, UNITED KINGDOM

## Abstract

**Purpose:**

To compare the effectiveness of an educational board game with interactive didactic instruction for teaching optometry students elements of the core optometric curriculum.

**Methods:**

Forty-two optometry students were divided into two GPA-matched groups and assigned to either 12 hours of game play (game group) or 12 hours of interactive didactic instruction (lecture group). The same material from the core optometric curriculum was delivered to both groups. Game play was accomplished via an original board game. Written examinations assessed change in knowledge level. A post-intervention opinion survey assessed student attitudes.

**Results:**

There was no significant difference in pre- or post-intervention test scores between the lecture and game groups (Pre-test: p = 0.9; Post-test: p = 0.5). Post-intervention test scores increased significantly from baseline (Game group: 29.3% gain, Didactic group: 31.5% gain; p<0.001 for each). The score increase difference between groups was not statistically significant (p = 0.6). The post-intervention attitude survey did not reveal any significant between group differences (p = 0.5).

**Conclusions:**

Our results indicate that an educational game and interactive didactic instruction can be equally effective in teaching optometry students basic and applied science. Furthermore, both modes of instruction have the potential to be equally engaging and enjoyable experiences.

## Introduction

An educational game is defined as an instructional method that requires the learner to participate in a competitive activity with preset rules[1]. There is a large body of literature reporting on the attitudes of medical and allied health students regarding the use of educational games in their training[2,3]. Almost invariably, students find educational games to be fun and enjoyable learning experiences. However, there is the potential for educational games to create stress or embarrassment among individuals who perform poorly[4].

Surveys have found that educational games are popular among teachers and students alike. The popularity of educational games in medical education is reflected in the finding that 80% of respondents to a survey of all Family Practice and Internal Medicine residency programs in the United States employ educational games in the training of their residents[5]. A recent survey of health educators in the United Kingdom found that teachers recognize that games are able to engage and motivate students[6]. Respondents to the survey reported that the main benefit of employing game play was enhancement of students’ learning, enjoyment, and interest.

Despite their popularity, systematic reviews of the literature find insufficient evidence to judge the effectiveness of educational games in the health professions, but available evidence suggests that they are not detrimental to the educational process[7–9]. While many papers have been published describing the use of educational games to teach health professionals, few are well-designed randomized controlled trials (RCT) which allow conclusions to be drawn regarding their effectiveness. A recent Cochrane review of the literature on educational games for the health professions found over 2000 papers published on the topic, but only two were well-designed RCTs with data that could be subjected to quantitative analysis[7]. The authors concluded that there is a need for more research of high methodological quality to explore the role of games in the education of health professionals.

Very little has been published on the use of games as a teaching tool in optometry. A search of the PubMed.gov database yielded only a single citation. The game described is a computer simulation to help optometry students learn test selection and lens prescribing skills[10]. The paper reported that 20 optometry students evaluated the game by playing a variety of simulated patients. The authors concluded that the game was a potentially useful educational tool. The purpose of this study was to evaluate the effectiveness of a board game of our own design in teaching optometry students elements of the optometric basic and applied science curriculum.

## Methods

This study was conducted in accordance with the principles of the Declaration of Helsinki. The protocol was approved by the Institutional Review Board at the University of the Incarnate Word. Subjects provided written informed consent after being appropriately informed of the nature of the study. This RCT was conducted during the 2014–2015 academic year at the Rosenberg School of Optometry in San Antonio, Texas, USA. It is reported in accordance with the CONSORT 2010 statement (consort-statement.org).

Research subjects were paid volunteers drawn from the student body of the Rosenberg School of Optometry (RSO). The inclusion criteria were that the subject be a third professional year optometry student (OD3) enrolled at RSO in good academic standing. Exclusion criteria included prior experience playing the game or students with a cumulative grade point average (GPA) of less than 2.0.

### Research Design

There were 2 study arms: experimental and control. Subjects were assigned to one of two groups solely on the basis of cumulative GPA, with the goal being to match the mean GPA of the groups as closely as possible. Following group assignment, the groups were designated as experimental or control by means of a coin toss. The control arm received 12 hours of interactive didactic instruction in the basic and applied sciences of the core optometric curriculum over a 2 consecutive day period (lecture group). The study arm played an educational board game covering the same material from the optometric curriculum for an equal number of hours over a 2 consecutive day period (game group). Baseline knowledge of the subject material was assessed immediately prior to the intervention with a 40-question multiple choice examination. Immediately following the intervention a different 40-question multiple choice examination was administered to assess change in knowledge level. Both groups received their instruction concurrently, with instructors rotating between the lecture and game groups on separate days. The examinations were administered to all subjects simultaneously prior to and after their game play or didactic instruction. The pre- and post-intervention exams administered to the experimental (game) group were identical to the pre- and post-intervention exams administered to the control (lecture) group.

Control arm subjects received interactive didactic instruction by the same instructors who participated in game play with the study arm subjects. Each instructor delivered a 3-hour lecture on a specific topic. The topics mirrored those covered in the game; specifically optics, basic science, vision science, and disease. The nature of the interactive instruction consisted of engaging the class in a variety of question-answer type activities. Most of the interactive activities included use of an audience response system, such as Turning Point® or Socrative,® but a variety of didactic activities were employed depending upon the style and preference of the instructor. The instructors specifically avoided passive lecturing to the control arm subjects.

The pre-test examination, post-test examination, and the content of the didactic instruction were each designed to provide a fair overview of the optometric basic and applied science curriculum within the constraints of a 40-question exam or a 3-hour lecture. Neither the lectures nor the exams were specifically designed to replicate the questions presented in the game. In other words, while every effort was made to ensure that the subject matter delivered to both groups was identical, no attempt was made to use identical questions.

### Educational Game Description

**Fig 1:**
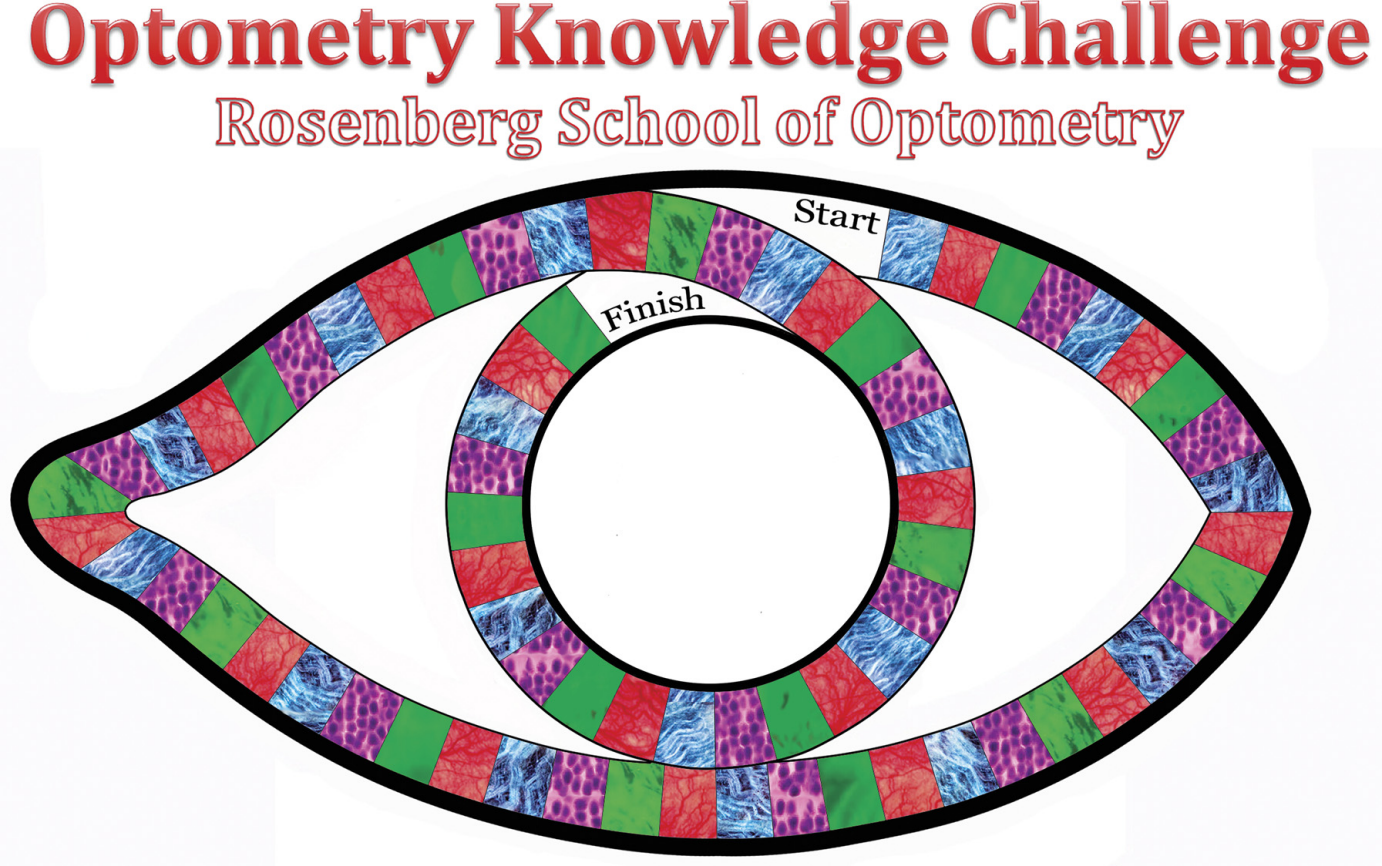
The Optometry Knowledge Challenge game board. The University logo has been removed from the center of the game board to comply with copyright requirements.

The educational game used in this study is one of our own design, which we have named the “Optometry Knowledge Challenge.” It is a board game wherein 2–6 players take turns, each rolling a die and moving his/her game piece a corresponding number of spaces around the board, accumulating points in the process (Fig 1). Play proceeds clockwise around the table. The first player to reach the end of the board receives bonus points and the game ends. The winner is the player with the greatest number of points at the end of the game. No rewards were offered to the winners in this study.

Each time a player moves his game piece he is asked an optometry-related question by a Judge, who is a faculty member that acts as a moderator for the game. Questions are in one of 4 topic areas: Basic Science, Vision Science, Optics, and Disease. After the player has moved the Judge draws a card containing questions from a deck of 150 cards. Each card contains 4 questions, one in each topic area, along with the correct answer. The questions used in this game are all short answer or fill-in-the-blank. All questions were written by the faculty and students of RSO, and were drawn from the core curriculum of the optometry school. The question topic is determined by the color of the space upon which the player’s game piece has landed. If the player answers the question correctly, he/she is awarded one point, and rolls the die a second time to advance his/her piece closer to the end of the board, but is not asked a second question. If the player answers incorrectly, no points are awarded and his/her turn ends immediately (he/she does not roll the die a second time). Players must answer questions from memory and are not allowed to consult notes or one another. The Judge is not allowed to offer hints or assist players in any other way in answering questions. Scratch paper and a calculator may be used by players, as needed. Players are allowed a maximum of 60 seconds to answer each question. The opinion of the Judge is final in the event of a dispute regarding whether the question has been answered correctly. After each question has been answered the Judge is free to explain the concept and entertain questions from the players.

To make play more exciting and interesting, 3 types of special cards are interspersed throughout the deck. The first is a “Go Back 3 Spaces” card. If this card is drawn, the player moves his game piece backward 3 spaces. A second card is drawn and the color of the space he lands upon after moving his piece backward determines the question topic. The second special card is “Move Forward 3 Spaces”. The third type of special card is the “Bonus” card. The questions on Bonus cards are more difficult and their point values are doubled.

When the end of the 150 card deck is reached the cards are shuffled and reused. This may result in the same question being asked in a subsequent game. This repetition is believed to enhance the learning process of the game because students who successfully recall answers from a previous game will perform better. Hence, students are motivated to attend to the questions and answers of their fellow players.

### Opinion Survey

At the conclusion of their participation in this study, subjects were asked to complete an opinion survey regarding their attitudes and perceptions of the instructional activity they received–either the didactic sessions or the game. The survey consisted of 6 statements that employed a 5-point Likert scale enabling subjects to express the degree to which they agreed or disagreed with statements pertaining to the enjoyment and educational merit of the activity (Table 1).

**Table 1:** Opinion survey statements.

Statement 1	Participating in this educational activity was fun
Statement 2	I learned a lot from participating in this educational activity
Statement 3	Participating in this educational activity was stressful
Statement 4	Participating in this educational activity motivated me to learn
Statement 5	I would like my instructors to offer educational activities similar to this
Statement 6	I enjoyed participating in this educational activity

### Statistical Analysis

Our research hypothesis was that the knowledge gain of the game group would be significantly different than that of the lecture group. As an exploratory study of the effectiveness of the Optometry Knowledge Challenge, we had no preexisting data upon which to conduct statistical power calculations. Therefore, our sample size was determined solely by the number of students that volunteered for our study. Student’s t-test was used to analyze pre- and post-intervention examination scores. Confidence intervals for the mean post-intervention change in exam scores for the study and control groups were calculated. The lower limit of the 90% confidence interval of the study group’s mean exam score change was used to assess equivalence of the intervention with the control group. Differences in opinion survey responses were evaluated with the Mann-Whitney U test and Kendall’s tau. The Mann-Whitney U test was used to test for significant difference in the distribution of responses to each question between the game and lecture groups. Associations in the survey data were investigated with Kendall’s tau.

Statistical analyses were carried out using Excel® 2010 (Microsoft, Redmond, WA), SPSS Statistics version 21 (IBM, Armonk, NY), and Prism 6 (GraphPad Software, La Jolla, CA). Post-hoc power analysis was conducted using G*Power® version 3.1.9.2[11]. Unless otherwise specified, data are presented as mean ± standard deviation.

## Results

A total of 42 subjects volunteered for the study (Table 2, S1 Data). All were eligible for participation; none were excluded, and all successfully completed the study. A total of 25 female and 17 male subjects participated in the study. Of the 21 subjects in the game group, 11 were female and 10 were male. In the lecture group there were 14 female and 7 male subjects. Female subjects had a slightly higher cumulative GPA than male subjects overall, and also in each of the study groups, but this difference never achieved statistical significance. The mean cumulative GPA for the game group was 3.19±0.45 and the lecture group was 3.19±0.45 (p = 1.0).

**Table 2:** Group demographics and exam scores*.

	N	GPA	Sex†	Baseline Score	Post-Test Score	Δ^§^	90% CI Δ
Game	21	3.19 (0.45)	11F, 10M	23.76 (4.85)	30.71 (3.38)	6.95 (3.37)	5.65–8.25
Lecture	21	3.19 (0.45)	14F 7M	23.90 (5.41)	31.43 (3.59)	7.52 (3.26)	6.27–8.78
p†		1		0.9	0.5	0.6	

GPA: Cumulative grade point average, F: Female, M: Male

*Expressed as mean (standard deviation)

†Student’s t-test, 2-tailed, α = 0.05

^§^Mean change in test scores

**Fig 2:**
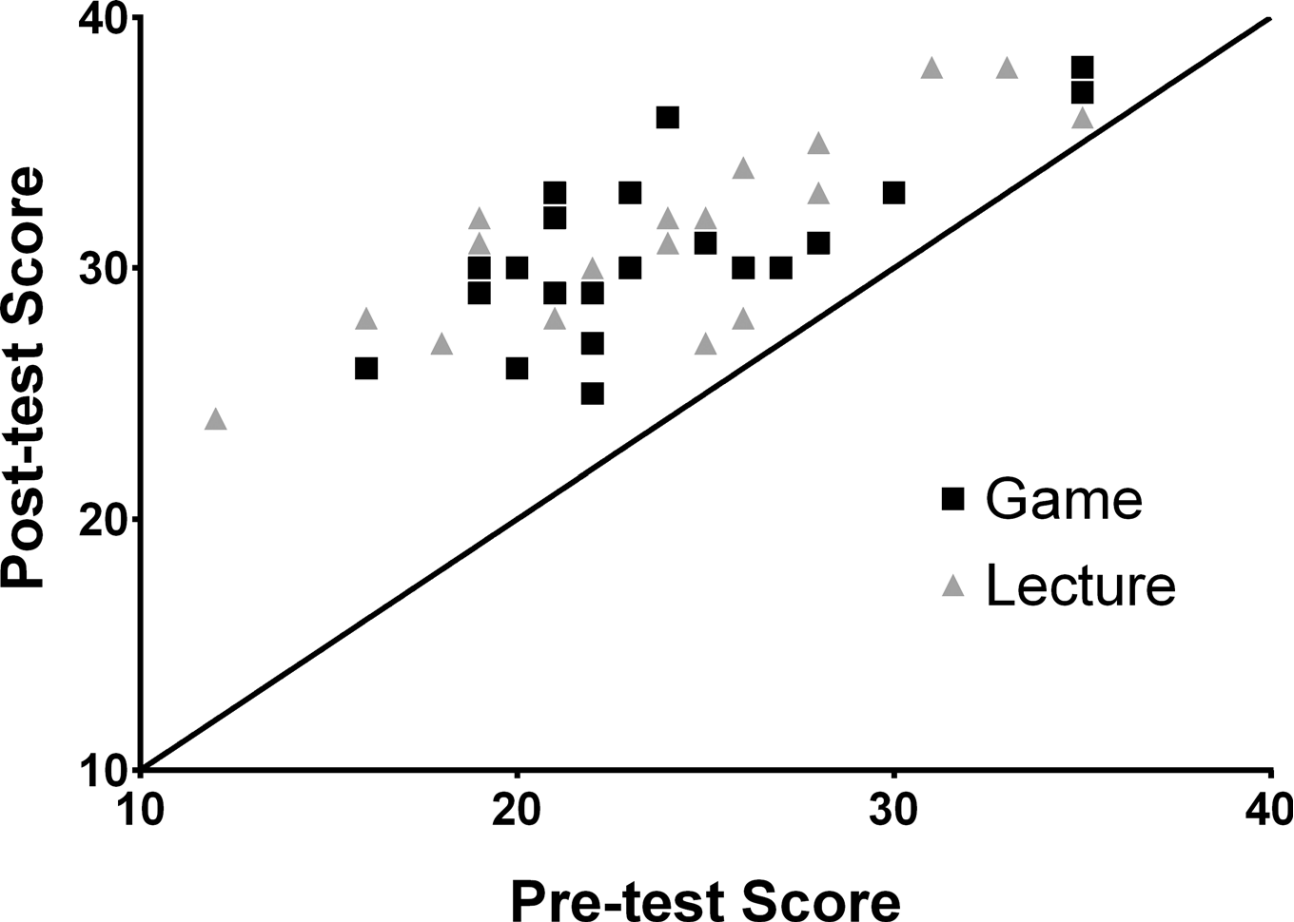
Scatter plot of pre-intervention and post-intervention raw exam scores for 42 study participants. The maximum possible score on each exam was 40 points. The solid line is the one-to-one line. If there was no change in score between the two exams, the data point would fall on the one-to-one line. Points above this line indicate an improvement in score following the intervention.

**Fig 3:**
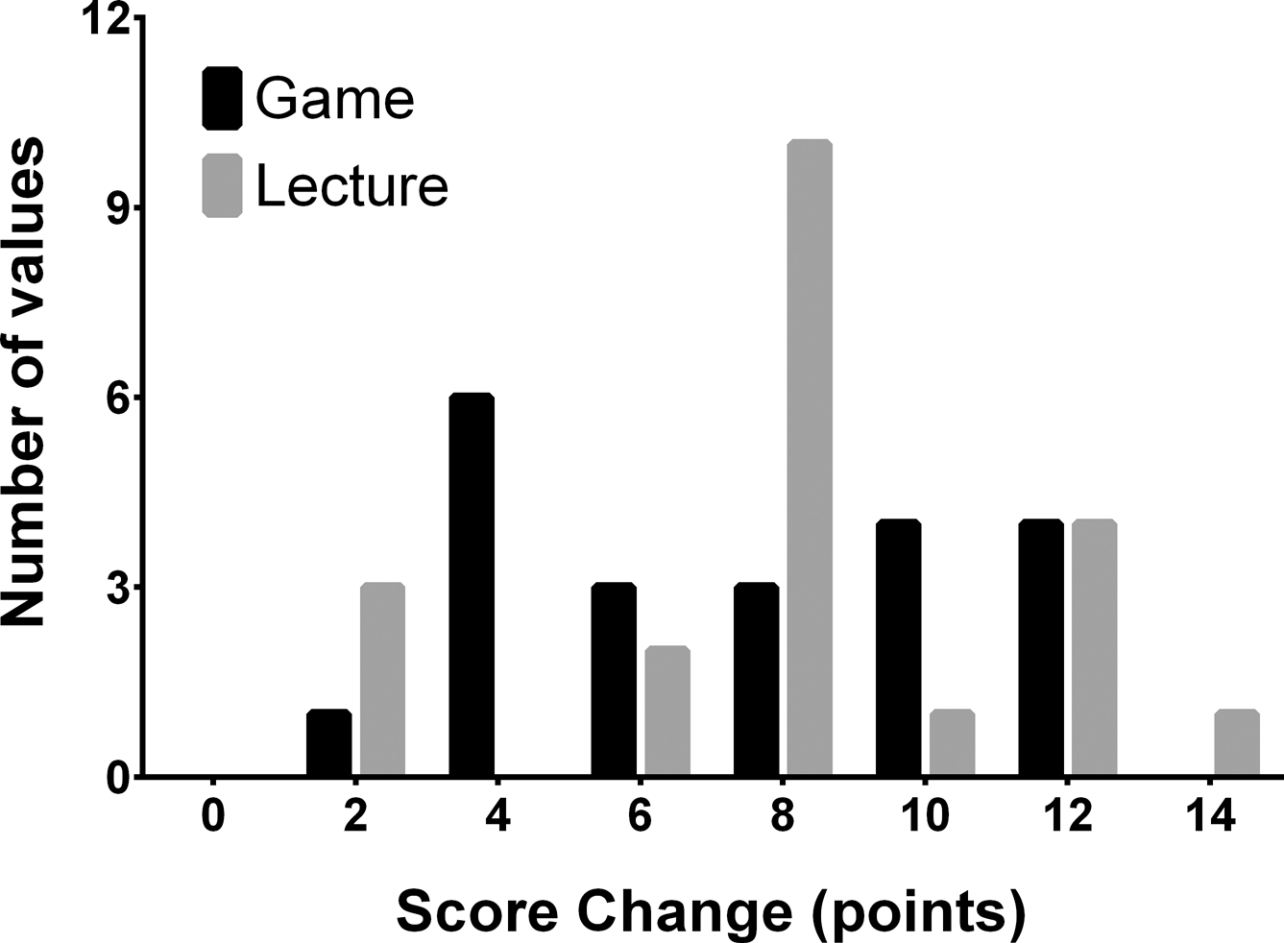
Frequency distribution of the pretest-posttest change in exam scores grouped by study arm. The mean change for the game group was 6.95±3.37 points and for the lecture group 7.52±3.26 points (p = 0.6). On average, participants with lower baseline scores experienced larger post-intervention gains.

The mean baseline examination score was 23.76±4.85 points for the game group and 23.90±5.41 points for the lecture group. There was a maximum possible score of 40 points on all examinations. The mean post-intervention examination scores were 30.71±3.38 points and 31.43±3.59 points for the game and lecture groups, respectively. There were no statistically significant differences between the groups in either baseline or post-intervention examination scores (baseline: p = 0.9; post-intervention: p = 0.5; Table 2). The improvement in exam scores from baseline was highly significant for both groups (Game group: 29.3% gain, Didactic group: 31.5% gain; p < 0.001 for each group; Figs 2 and 3). Participants in the bottom tertile (bottom 1/3) of baseline scores of each group had significantly larger gains on the post-intervention exam than participants in the top tertile (p < 0.001 for each group). There were no significant differences in tertile score gains between groups (p = 0.4 for bottom tertile, p = 0.2 for top tertile).

**Fig 4:**
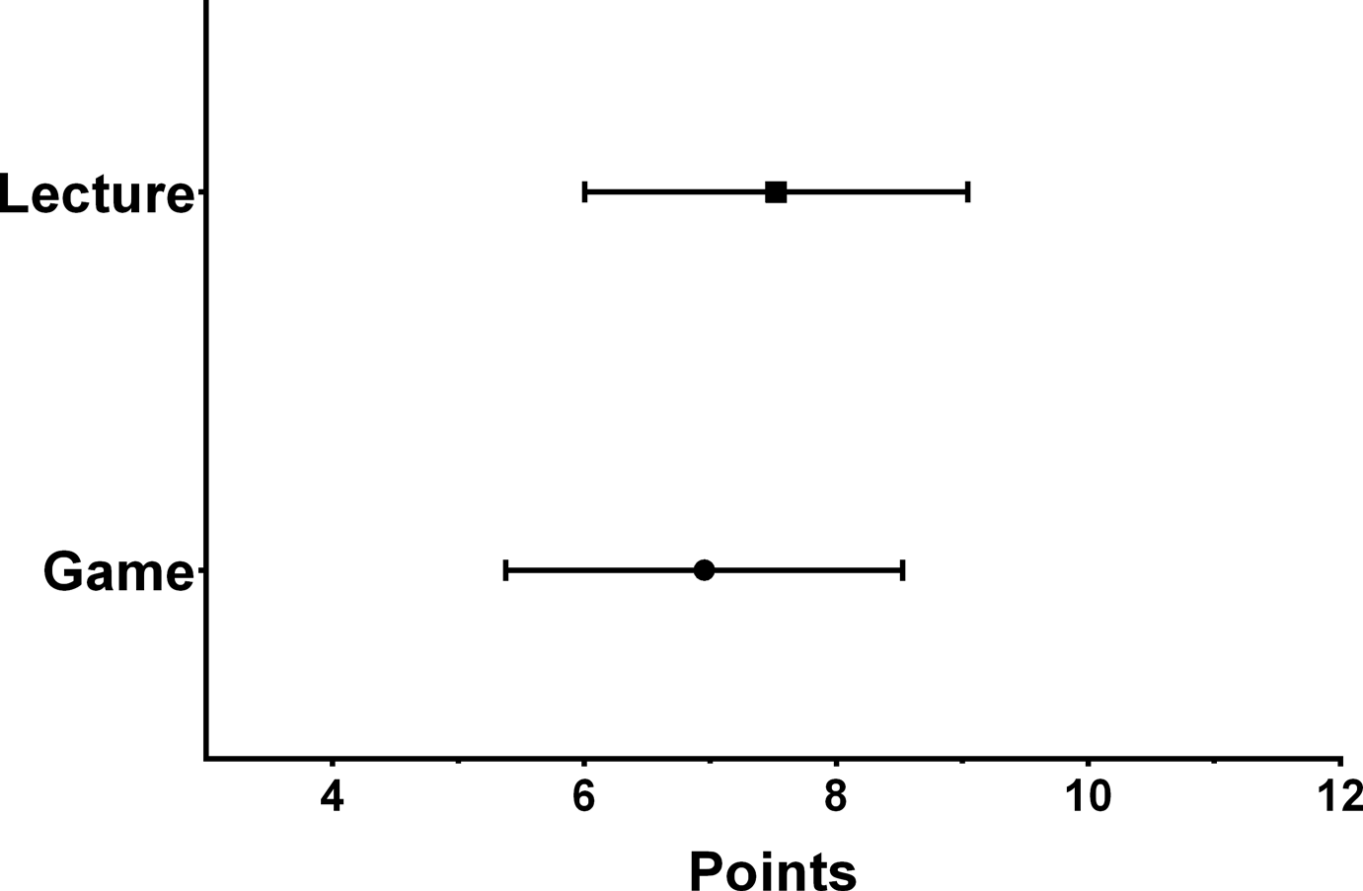
Mean change in exam score of the study and control groups following the intervention. Error bars represent the 95% confidence interval of the mean. The lower limit of the 90% CI of the mean change of the game group is 1.87 points lower than the mean change of the lecture group.

Because the mean exam score change of the game group was not significantly different than the mean change of the lecture group (p = 0.6), we are unable to reject our null hypothesis that the game and lecture are equally effective. We therefore analyzed the equivalence of the two interventions. The mean change from baseline for the game group was 6.95 points (90% confidence interval [CI]: 5.65–8.25), and for the lecture group the mean change was 7.52 (90% CI: 6.27–8.78). Because the lower limit of the 90% CI for the mean change of the game group is 1.87 points lower than the mean change of the lecture group (7.52–5.65), we have 95% confidence that the mean improvement for the game group is no more than 1.87 points less than the mean improvement of the lecture group (Fig 4). A score difference of 1.87 points is equivalent to 4.7% on a 40 question exam.

Two post hoc subgroup analyses were undertaken to investigate whether certain subpopulations of our subjects fared better with the game or didactic intervention. Subjects were analyzed by GPA (top 50% vs. bottom 50%) and by sex (Table 3, Table 4). The high GPA group performed significantly better than the low GPA group on both baseline and post-intervention examinations. This concordance between cumulative GPA and performance on our examinations provides evidence that the exams are valid assessment instruments. Subjects with high GPA status demonstrated less post-intervention gain in exam scores than low GPA status subjects, but this only achieved statistical significance among the lecture group (game: p = 0.2; lecture: p = 0.02). The mean point gain for high GPA subjects was nearly identical for each intervention (game: 5.80±3.22; lecture 5.80±2.40, p = 1.0), but the low GPA subjects saw a mean 1 point larger gain in the lecture group than the game group (game: 8.00±3.16; lecture: 9.09±3.15, p = 0.4). We uncovered no exam performance differences between male and female subjects between the two arms of our study.

**Table 3:** Analysis of score change by GPA subgroup*.

		N	GPA	Baseline Score	Post-Test Score	Score Change		p†
Game								
	High GPA	10	3.59 (0.23)	27.00 (4.69)	32.80 (3.03)	5.80 (3.22)		0.2
	Low GPA	11	2.81 (0.22)	20.82 (2.59)	28.82 (2.44)		8.00 (3.16)	
Lecture								
	High GPA	10	3.60 (0.22)	28.10 (3.53)	33.90 (3.08)	5.80 (2.40)		0.02
	Low GPA	11	2.82 (0.25)	20.09 (3.73)	29.18 (2.33)		9.09 (3.15)	
p†						1.0	0.4	

GPA: Cumulative grade point average.

*Expressed as mean (standard deviation)

†Student’s t-test, 2-tailed, α = 0.05.

**Table 4:** Analysis of score change by sex subgroup*.

		N	GPA	Baseline Score	Post-Test Score	Score Change		p†
GAME								
	Female	11	3.32 (0.47)	24.45 (4.36)	31.82 (3.33)	7.36 (3.57)		0.6
	Male	10	3.06 (0.40)	23.00 (5.23)	29.50 (3.01)		6.50 (3.07)	
LECTURE								
	Female	14	3.20 (0.52)	23.64 (6.33)	31.36 (4.12)	7.71 (3.49)		0.7
	Male	7	3.17 (0.27)	24.43 (2.66)	31.57 (2.19)		7.14 (2.70)	
p†						0.8	0.7	

GPA: Cumulative grade point average, F: Female, M: Male

*Expressed as mean (standard deviation)

†Student’s t-test, 2-tailed, α = 0.05.

**Fig 5:**
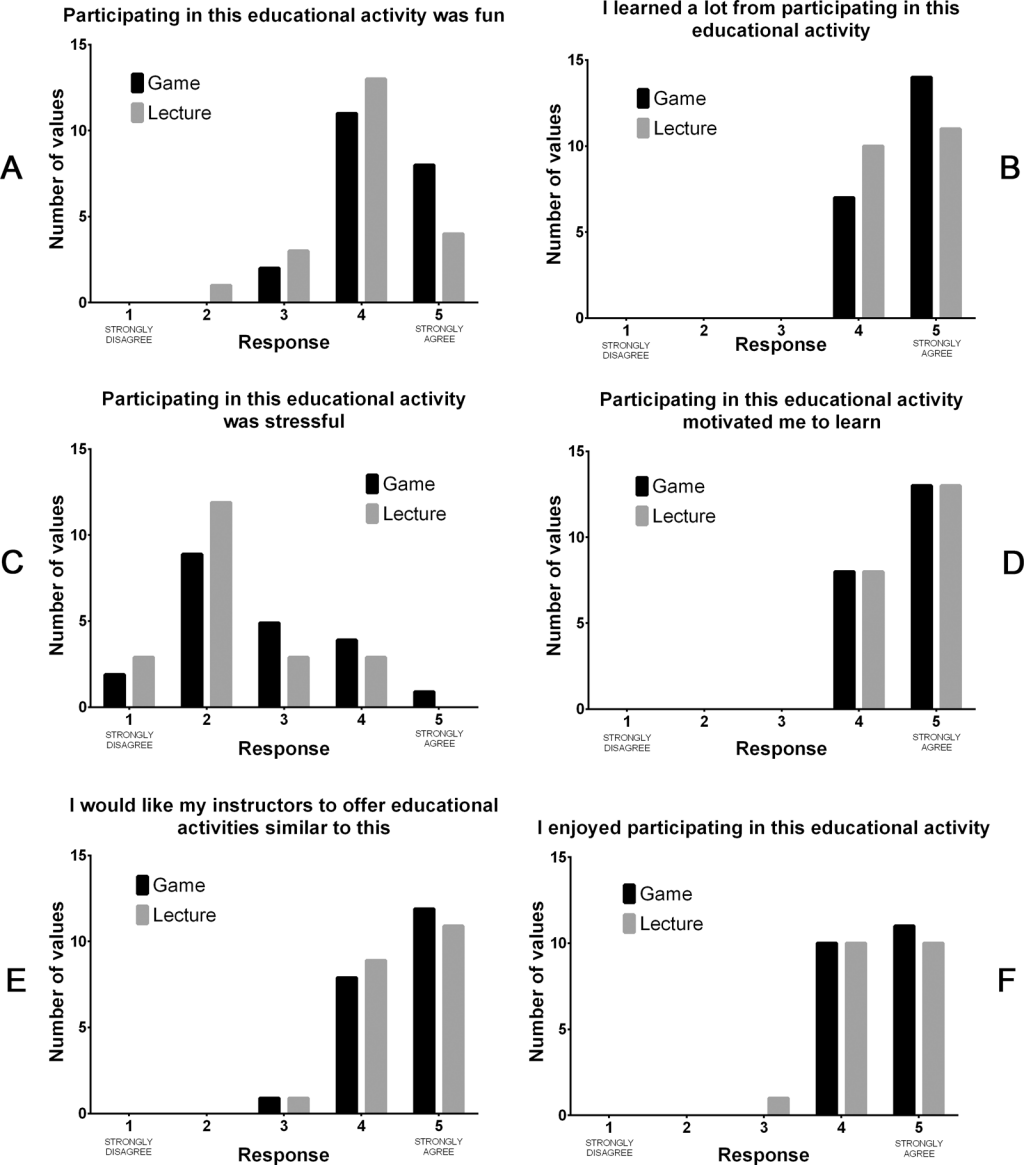
Frequency distribution of responses to the post-intervention opinion survey grouped by study arm. Participants were asked to indicate the degree to which they agreed or disagreed with each of the 6 statements using a 5-point Likert scale, with 1 indicating “Strongly Disagree” and 5 indicating “Strongly Agree”.

Following the intervention, subjects expressed their level of agreement with 6 statements pertaining to the enjoyment, educational merit, and stress of the interventions using a 5-point Likert scale (Table 1, Fig 5). The Mann-Whitney U test did not reveal any significant differences in the distribution of responses between the game and lecture groups for any of the 6 statements (Table 5). We also analyzed the survey responses using the Kendall tau-b test to reveal associations between responses to pairs of statements within each experimental group (Table 6). Of note is the finding that there were no correlations between the degree to which the subject felt stressed (Statement 3) and their responses to any other statement in either group.

**Table 5:** Analysis of opinion survey responses.

Statement		U Statistic*	P†
1	Participating in this educational activity was fun	168.500	0.141
2	I learned a lot from participating in this educational activity	189.000	0.351
3	Participating in this educational activity was stressful	175.500	0.223
4	Participating in this educational activity motivated me to learn	220.500	1.000
5	I would like my instructors to offer educational activities similar to this	210.500	0.774
6	I enjoyed participating in this educational activity	205.000	0.656

*Mann-Whitney U statistic

†2-tailed, α = 0.05

**Table 6:** Correlation of opinion survey responses*.

		Statement 2	Statement 3	Statement 4	Statement 5	Statement 6
Statement 1						
	Game	0.005	0.107	0.026	0.019	0.001
	Lecture	0.035	0.532	0.532	0.474	0.014
Statement 2						
	Game		0.875	0.002	< 0.001	0.001
	Lecture		0.724	0.867	0.231	0.015
Statement 3						
	Game			0.970	0.659	0.236
	Lecture			0.183	0.317	0.619
Statement 4						
	Game				0.001	0.005
	Lecture				0.218	0.902
Statement 5						
	Game					0.001
	Lecture					0.016

*Expressed as the 2-tailed significance value of Kendall's tau-b correlation coefficient.

## Discussion

We found that an educational board game of our own design was not significantly different than an interactive didactic experience in increasing knowledge of basic and applied science among OD3 students. Furthermore, there was no significant difference in how our subjects rated their enjoyment and educational merit of their experiences. These results add to the growing body of evidence that educational games are an effective instructional tool for the health professions.

Our game was designed as a tool to assist OD3 students in the course of their preparations to take the Applied Basic Science examination administered by the National Board of Examiners in Optometry (www.optometry.org). As such, it is intended to serve as a review of material they have already been exposed to within the optometric curriculum. We purposefully recruited OD3 students as subjects to simulate this review experience but timed our study to take place prior to the students commencing their board preparation efforts to avoid contamination of our results by prior review work on the part of the students. As such, our results should be interpreted within the context of a review exercise rather than introducing students to material which is completely foreign to them. The value of educational games as a method of reviewing and reinforcing key concepts in medical education has been previously noted [12,13]. Indeed, review and reinforcement of student’s knowledge and understanding seems to be the most popular and most appropriate use of educational games [14].

Selection of an appropriate control group is of utmost importance in designing a research study. Akl, et al. in their systematic review of the use of educational games in medical education concluded that the ideal control intervention is an interactive lecture because it represents the best available alternative to game play [2]. They recommended that the control and experimental interventions be made as similar as possible, including, for example, having the duration of the interventions and the persons administering them held constant. Under such conditions only the game play itself would be responsible for any observed outcome differences between the groups. In designing this study we made every effort to ensure that our groups were well matched and that wherever possible the interventions were identical. The two groups were evenly matched in terms of cumulative GPA and had similar numbers of male and female subjects. The content, duration, and faculty of interventions were identical. The control intervention was specifically designed to be as engaging and interactive as possible in a didactic setting, with most instructors using an audience response system for some or all of their presentation. Mains et al. demonstrated that use of an audience response system improves learning and retention over traditional lectures [15]. Therefore, we believe that our results are an accurate indication of the effectiveness of an educational game relative to the best available alternative.

In their recent review of the use of educational games in the health professions, Abdulmajed, Park and Tekian emphasized the need for reliable and valid tools for assessing their effectiveness [9]. They pointed out that existing assessment methodologies are not capable of fully capturing the richness of the learning that may be occurring in these games. For example, their potential impact on long-term memory and behavior in a clinical setting. There is currently no consensus with regard to the optimal method of assessing game-based learning [16]. Our assessment tool was a very traditional multiple choice examination. Correlation between the participant’s cumulative GPA and performance on the baseline examination suggests that this assessment tool is a valid measure of their knowledge level, and that gains seen on the post-intervention examination accurately reflect short-term changes in their knowledge level. However, other dimensions of learning that may be occurring are not reflected in the results of our study. It has been suggested that educational games promote learning in the cognitive, affective, and psychomotor domains [7,17,18]. Therefore, the potential value of educational games in the health professions should not be judged solely on the basis of their effect on short-term knowledge gains.

Our results suggest that playing the educational game had an equivalent effect on knowledge level as participation in an interactive didactic lecture. We determined with 95% confidence that the mean exam score improvement of the game group was no more than 4.7% less than that of the lecture group (Table 2). This difference of 1.87 points is quite small in light of the significant variability of the observed mean score changes (the SD of the mean change was 3.37 and 3.26 in the game and lecture groups, respectively). Perhaps with larger sample sizes future studies can determine the relative effectiveness of education games with greater precision. Within the context of this study, however, a difference of this magnitude is likely within the margin of measurement error. Therefore, we conclude that our findings demonstrate the non-inferiority of the educational game relative to the interactive didactic experience.

Our post-hoc subgroup analysis revealed an interesting difference in the performance of low GPA students (Table 3). While low GPA students achieved larger gains than their high GPA counterparts in both the game and lecture groups, this difference was only statistically different in the lecture group. This may be related to the fact that the mean difference between the high GPA and low GPA students was larger at baseline in the lecture group than in the game group (game: 6.18 points, lecture: 8.01 points). However, it is interesting to speculate that it may be related to differences in learning styles. This possibility is supported by the finding that the low GPA students in the lecture group scored 0.36 points higher on the post-intervention exam than the low-GPA students in the game group despite being 0.73 points lower at baseline. While our sample sizes are too small to make any meaningful statistical inferences of these subgroup comparisons, we suggest that future studies investigate this further.

While there have been many studies published on the topic of educational games in the training of health care professionals, there are very few comparable RCTs against which to compare our findings. Comparable studies would be those that employed interventions that are similar to ours. Most other RCTs on this subject are not directly comparable because there was either no control intervention or it was a conventional lecture. The control intervention in our study was an interactive lecture, which is specifically designed to be more engaging than a conventional didactic lecture. Furthermore, we should compare our findings to studies that employed a similar experimental intervention. Our experimental intervention being a board game could be compared to other similar types of games, such as other board games, card games, and other similar activities. Computer-based games, in general, would not be comparable. Finally, our outcome measure was change in knowledge level that was assessed immediately following the intervention. Comparable studies would have done the same.

We have identified a single published study that meets all of the above criteria. Selby and colleagues [19] conducted a RCT comparing an interactive lecture with a game based on charades as a means of teaching child development to 100 fifth year medical students. Post-intervention knowledge level was assessed by means of a quiz immediately following the intervention. No baseline knowledge level assessment was performed. Students in the lecture group performed significantly better on the post-intervention quiz than students in the game group, but the effect size was small (p < 0.01, r = 0.23). The investigators concluded that an interactive lecture can improve short-term retention of knowledge more than a game.

There are a number of additional studies that meet some but not all of our criteria for study equivalence. For example, Montpas et al. compared the use of a Jeopardy®-type game versus conventional lecture on 68 nursing students' achievement and retention of geriatric nursing concepts [20]. Students were randomized into treatment and control groups. Baseline knowledge was assessed using a pretest, and knowledge gains were assessed with a post-test that was administered immediately following the intervention. Results showed that lecture was statistically more effective than gaming in short-term knowledge gains; however, the game group performed better than the lecture group in a reassessment that was performed 2 weeks later. Another RCT of a Jeopardy®-type game versus conventional lecture was undertaken by O’Leary and colleagues [21]. This study investigated knowledge gain of obstetrics and gynecology among 104 third year medical students. A pretest was conducted before the intervention and a post-test measured knowledge gain immediately afterward. Despite the lecture group performing significantly worse on the pretest, the two groups had nearly identical mean post-test scores.

Our results are in line with these previous studies in finding no clear superiority of either game play or didactic instruction in generating short-term knowledge gains. Both forms of instruction seem equally capable of generating significant short-term increases in knowledge level.

Interestingly, we found no significant difference in rating of enjoyment, engagement and perceptions of educational merit between our two interventions (Table 5). Previous studies comparing games to conventional lecture find large and consistent differences, with games being consistently rated as being more enjoyable. O’Leary et al., for example, reported that students participating in the game rated it higher in stimulating faculty/student interaction, helping them to retain information, and enjoyment (p < 0.001)[21]. Because Selby, et al. [19] did not investigate student opinions and attitudes, we are not aware of a RCT other than ours which directly compares game play with interactive lecture in the education of healthcare professionals. Interactive lectures share many of the purported advantages of educational games, including fostering an active learning environment, improving engagement, and increasing comprehension and retention of material [15]. Therefore, it may not be surprising that we did not uncover a clear student preference for one instruction method over the other.

There are conflicting reports regarding whether educational games are more stressful than conventional instructional methods. It has been reported that some students find educational games to be stressful or uncomfortable because of embarrassment at giving incorrect answers to questions; while other investigators report that playing games can reduce classroom stress and anxiety [2,8,9]. We did not find any significant difference in the responses of game and lecture group participants to the statement “Participating in this educational activity was stressful” (Table 5). While a majority of subjects in both groups disagreed with that statement, there were more participants in the game group that agreed or strongly agreed with the statement than lecture group subjects (Fig 5C). Additionally, we found no correlation between student perception of stress and their ratings of enjoyment or educational merit for either educational intervention (Table 6). Therefore, it seems that students who reported more stress did not necessarily find the experience to be less enjoyable or to have gotten less out of it.

Strengths of this study include the close GPA matching of the control and experimental groups, the use of interactive lecture as the control intervention, and the administration of both baseline and post-intervention assessments of knowledge level. Our study has some important weaknesses that need to be considered when interpreting our results. Our sample size was relatively small, making statistical evaluation of the small differences between our interventions challenging. The effect size of the difference in knowledge gain between the study and control groups was small (d = 0.18). Therefore our achieved power was only 10% (α = 0.05, two-sided t-test). To achieve 80% power would require each group to contain 486 subjects. Another possible weakness was that the entire 12 hour intervention for each group was completed over the course of 2 consecutive days. Therefore, by the end of the second day when the post-test and opinion survey were being completed, the students may have been fatigued and not in the best frame of mind to be rating their opinions and perceptions of the intervention.

In summary, we found no significant difference between an educational board game and interactive didactic instruction on short-term knowledge gains. Furthermore, both interventions were rated as being equally enjoyable, engaging, and educationally valuable by the students. We conclude that the Optometry Knowledge Challenge game is non-inferior to interactive didactic instruction. Future studies are needed to determine whether specific subgroups of individuals, including those with lower academic achievement level or specific learning styles, may benefit more from engagement in educational games. To our knowledge, this is the first study to test the effectiveness of educational games in a School or College of Optometry.

## Supporting Information

S1 Data Exam and Opinion Survey Data. (XLSX)
